# Manogepix demonstrates potent *in vitro* activity against *Aspergillus, Coccidioides*, *Scopulariopsis/Microascus, Rasamsonia*, and *Purpureocillium* species, including strains with *in vitro* resistance to other antifungals

**DOI:** 10.1128/aac.00476-26

**Published:** 2026-06-04

**Authors:** Nathan P. Wiederhold, Hoja P. Patterson, Carmita Sanders, Connie Canete-Gibas

**Affiliations:** 1 Fungus Testing Laboratory, Department of Pathology and Laboratory Medicine, University of Texas Health Science Center at San Antonio 14742, San Antonio, Texas, USA; University of Iowa, Iowa City, Iowa, USA

**Keywords:** manogepix, antifungal activity, filamentous fungi

## Abstract

Manogepix, the active moiety of the prodrug fosmanogepix, is a novel investigational agent in clinical trials that inhibits Gwt1, depleting glycosylphosphatidylinositol (GPI)-anchored mannoproteins critical for fungal cell wall integrity. It has potent broad-spectrum antifungal activity, including against fungi that are often resistant to clinically available antifungals (e.g., *Fusarium* species and *Lomentospora prolificans*). Our objective was to evaluate the *in vitro* activity of manogepix against different *Aspergillus* species, *Coccidioides,* and rare molds with reduced *in vitro* susceptibility or resistance to other antifungals. A total of 257 clinical isolates of *Aspergillus, Coccidioides, Scopulariopsis/Microascus*, *Rasamsonia argillaceae* species complex members, *Purpureocillium lilacinum, Curvularia, Exophiala,* and *Exserohilum rostratum* were used. Minimum effective concentrations (MECs) (manogepix and caspofungin) and minimum inhibitory concentrations (MICs) (amphotericin B, fluconazole, isavuconazole, itraconazole, posaconazole, and fluconazole) were determined by CLSI M38 broth dilution. Manogepix demonstrated potent *in vitro* activity against *Aspergillus, Coccidioides,* and several other molds (MEC range ≤ 0.002–0.015 mg/L; GM MEC range 0.003–0.007 mg/L). The activity of manogepix was also maintained against strains with reduced *in vitro* susceptibility to, or resistance to, other antifungals, including those with elevated MEC values to caspofungin, as well as isolates that were intrinsically resistant to other antifungals. Reduced or variable activity for manogepix was observed against *Curvularia, Exophiala,* and *Exserohilum rostratum*. Additional studies are warranted to determine if this potent *in vitro* activity translates into *in vivo* activity and enhanced clinical efficacy against infections caused by these fungal pathogens.

## INTRODUCTION

Over the last two decades, progress has been made in the treatment of invasive aspergillosis, one of the most prevalent invasive mold infections, with the availability of the extended-spectrum triazoles, voriconazole, posaconazole, and isavuconazole (1–3). However, there is now increasing concern regarding *Aspergillus* infections caused by azole-resistant strains of *A. fumigatus* (4–6), as well as those caused by less common cryptic species with reduced susceptibility or resistance to available antifungals, including *A. lentulus* and *A. calidoustus,* among others (7–10). In addition, there are limited treatment options against less common molds that may display reduced *in vitro* susceptibility to or intrinsic resistance to different antifungals. These include members of the genera *Microascus* and *Scopulariopsis,* those of the *Rasasmonia argillaceae* species complex, and *Purpureocillium lilacinum*, the latter two of which are intrinsically resistant to voriconazole/isavuconazole and amphotericin B, respectively (11–14). Finally, there is also concern about the expanded endemic area of the dimorphic *Coccidioides* species, *C. immitis* and *C. posadasii,* within the Western hemisphere, along with reduced *in vitro* susceptibility to fluconazole in these fungi (15–17). Thus, there is a need for the continued development of broad-spectrum antifungal agents, including those that maintain activity against fungi that are resistant to clinically available drugs.

Manogepix is a novel antifungal that targets the enzyme Gwt1, an inositol acyltransferase involved early in the glycosylphosphatidylinositol (GPI)-anchored protein maturation pathway (18–20). GPI-anchored mannoproteins are important for adherence of fungi to host cell surfaces needed for colonization and infections (18, 19, 21). Studies have reported that manogepix has broad-spectrum and potent *in vitro* activity against numerous pathogenic species, including both yeasts and molds, which is maintained against isolates that are resistant to clinically available antifungals (20). *In vivo* activity has also been reported in different animal models of invasive fungal infections (22–33), and efficacy has also been reported in small clinical studies to date (34–36). However, *in vitro* data against rare molds, including cryptic species of *Aspergillus*, and others with reduced *in vitro* susceptibility or resistant to clinically available antifungals, are limited or unknown. Our objective was to evaluate the *in vitro* activity of manogepix against *Aspergillus* species, including azole-resistant strains, rare molds that have reduced susceptibility or resistance to different antifungals, and *Coccidioides* species.

## RESULTS

### Aspergillus

Overall, manogepix demonstrated potent *in vitro* activity against all *Aspergillus* strains included in this study, regardless of species and susceptibility to other antifungals. The overall results and minimum inhibitory concentration (MIC)/minimum effective concentration (MEC) distributions for each antifungal against all 72 strains within this genus are shown in Table 1, and results against different sections and specific species are shown in Table 2. Both manogepix and caspofungin demonstrated potent *in vitro* activity against all strains within section *Fumigati*, including those of *A. fumigatus sensu stricto* and *A. lentulus,* the latter of which had higher voriconazole MIC values. In addition, manogepix and caspofungin both maintained activity against six *A. fumigatus* strains that were resistant to voriconazole and/or posaconazole. This included those that harbored TR_34_/L98H (*n* = 2) and TR_46_/Y121F/T289A (*n* = 3) mutations within Cyp51A (manogepix MECs 0.015 mg/L vs all; caspofungin MECs 0.03–0.125 mg/L).

**TABLE 1:** MEC/MIC parameters and distributions (number of strains at each MIC/MEC value) against all *Aspergillus* strains (*n* = 72)^*a*^

Parameter	Manogepix (MEC)	Caspofungin (MEC)	Voriconazole (MIC)	Posaconazole (MIC)										
Range	≤0.002–0.03	0.004–2	0.06 to >16	≤0.03 to >16										
MEC/MIC_50_	0.008	0.03	1	0.25										
MEC/MIC_90_	0.015	1	8	32										
GM MEC/MIC	0.008	0.039	1.37	0.333										
Modal MEC/MIC	0.015	0.015	1	0.25										
MEC/MIC distributions														
MEC/MIC (mg/L)	≤0.002	0.004	0.008	0.015	0.03	0.06	0.12	0.25	0.5	1	2	4	8	≤16
Manogepix (MEC)	9	10	23	27	3									
Caspofungin (MEC)		1	2	27	21	10	3	5	3					
Voriconazole (MEC)						1	3	9	9	20	11	3	12	4
Posaconazole (MIC)					7	15	7	19	10	3	1			10

^*a*^ All values reported in mg/L.

**TABLE 2:** MEC/MIC parameters against different *Aspergillus* sections and specific species^*a*^

Antifungal	Manogepix (MEC)	Caspofungin (MEC)	Voriconazole (MIC)	Posaconazole (MIC)
*Aspergillus* section *Fumigati* (*n* = 20)				
Range	0.008–0.015	0.015–0.125	0.125 to >16	≤0.03 to 2
MEC/MIC_50_	0.015	0.06	2	0.25
MEC/MIC_90_	0.015	0.125	>16	1
GM MEC/MIC	0.011	0.040	2.64	0.257
Modal MEC/MIC	0.015	0.06	1	0.25
*Aspergillus fumigatus* (*n* = 10)				
Range	0.015	0.015–0.125	0.125 to >16	≤0.03 to 2
MEC/MIC_50_	0.015	0.06	2	0.25
MEC/MIC_90_	0.015	0.125	>16	1
GM MEC/MIC	0.015	0.049	2.83	0.26
Modal MEC/MIC	0.015	0.06	>16	0.03
Azole-resistant *A. fumigatus* strains (*n* = 6)				
Range	0.015	0.03–0.125	2 to >16	0.25–2
*Aspergillus lentulus* (*n* = 10)				
Range	0.008–0.015	0.015–0.06	1 to >16	0.125–0.5
MEC/MIC_50_	0.008	0.03	2	0.25
MEC/MIC_90_	0.015	0.06	>16	0.5
GM MEC/MIC	0.009	0.032	2.46	0.25
Modal MEC/MIC	≤0.008	0.06	1	0.25
*Aspergillus* section *Nigri* (*n* = 13)				
Range	≤0.002–0.008	0.004–0.03	1–2	0.06–0.5
MEC/MIC_50_	0.002	0.015	1	0.25
MEC/MIC_90_	0.004	0.03	2	0.5
GM MEC/MIC	0.003	0.016	1.38	0.277
Modal MEC/MIC	≤0.002	0.015	1	0.5
*Aspergillus niger* (*n* = 3)				
Range	≤0.002–0.004	≤0.015–0.03	1	0.06–0.125
*Aspergillus tubingensis* (*n* = 10)				
Range	≤0.002–0.004	0.015–0.03	1–2	0.25–0.5
MEC/MIC_50_	≤0.002	0.015	2	0.5
MEC/MIC_90_	0.004	0.03	2	0.5
GM MEC/MIC	0.003	0.015	1.52	0.379
Modal MEC/MIC	≤0.002	0.03	2	0.5
*Aspergillus* section *Terrei* (*n* = 13)				
Range	0.008–0.03	0.015–0.125	0.06–2	≤0.03–0.06
MEC/MIC_50_	0.008	0.015	0.25	0.06
MEC/MIC_90_	0.015	0.06	0.5	0.06
GM MEC/MIC	0.011	0.023	0.343	0.051
Modal MEC/MIC	0.008	0.015	0.25	0.06
*Aspergillus terreus* (*n* = 3)				
Range	0.015–0.03	≤0.015–0.03	0.5–2	0.06
*Aspergillus hortai* (*n* = 10)				
Range	0.008–0.015	0.015–0.125	0.06–0.5	≤0.03–0.06
MEC/MIC_50_	0.008	0.015	0.25	0.06
MEC/MIC_90_	0.015	0.125	0.5	0.06
GM MEC/MIC	0.010	0.024	0.267	0.049
Modal MEC/MIC	0.008	0.015	0.25	0.06
*Aspergillus sydowii* (*n* = 10)				
Range	≤0.008–0.03	0.015–0.06	0.25–2	0.06–0.5
MEC/MIC_50_	0.015	0.03	1	0.25
MEC/MIC_90_	0.03	0.03	1	0.25
GM MEC/MIC	0.014	0.023	0.812	0.175
Modal MEC/MIC	0.015	0.015	1	0.25
*Aspergillus calidoustus* (*n* = 10)				
Range	≤0.002–0.008	0.015–2	8	>16
MEC/MIC_50_	0.004	1	8	>16
MEC/MIC_90_	0.008	2	8	>16
GM MEC/MIC	0.005	0.570	8	>16
Modal MEC/MIC	0.008	1	8	>16
*Aspergillus flavus* (*n* = 3)				
Range	0.008	0.015	1	0.125–0.25

^*a*^ All values reported in mg/L.

MEC results for manogepix and caspofungin against species within other *Aspergillus* sections were similar to what was observed against members of section *Fumigati*. Of note, manogepix also maintained potent activity against *A. calidoustus* isolates (MEC values ≤ 0.002–0.008 mg/L, modal MEC 0.008 mg/L). In contrast, MEC values for caspofungin were elevated (0.015–2 mg/L, modal MEC 1 mg/L), and the GM MEC of this echinocandin (0.570 mg/L) was significantly higher than that of manogepix (0.005 mg/L; *P* < 0.0001). Voriconazole MIC values were higher against this species (8 mg/L) compared to the other *Aspergillus* species, and posaconazole demonstrated no *in vitro* activity (>16 mg/L vs all strains).

### Coccidioides

Results against *Coccidioides* spp., including *C. immitis* and *C. posadasii* strains, are shown in Table 3. Manogepix demonstrated potent *in vitro* activity with MEC values ranging from ≤0.002–0.03 mg/L. MIC values for amphotericin B were also low, as were the majority of those for itraconazole and isavuconazole. MIC values were higher for fluconazole (range 2–64 mg/L). Manogepix also maintained activity (MEC 0.015 mg/L) against the single isolate included that had very high fluconazole (64 mg/L) and itraconazole (>16 mg/L) MIC values.

**TABLE 3:** MEC/MIC parameters and distributions against *Coccidioides* spp. strains (*n* = 53)^*a*^

Parameter	Manogepix (MEC)	Amphotercin B (MIC)	Fluconazole (MIC)	Itraconazole (MIC)	Isavuconazole (MIC)											
Range	≤0.002–0.03	≤0.03–0.5	≤0.03–64	≤0.03 to >16	≤0.03 to 2											
MEC/MIC_50_	0.008	0.06	8	0.06	0.25											
MEC/MIC_90_	0.015	0.25	16	0.25	0.5											
GM MEC/MIC	0.007	0.058	6.16	0.095	0.308											
Modal value	0.008	0.03	8	0.06	0.5											
MEC/MIC distributions																
MEC/MIC (mg/L)	0.002	0.004	0.008	0.015	0.03	0.06	0.125	0.25	0.5	1	2	4	8	16	32	64
Manogepix	2	12	28	10	1											
Amphotericin B					25	13	10	3	2							
Fluconazole											3	21	24	4		1
Itraconazole					9	18	16	9								
Isavuconazole					1		9	19	21	2	1					

^*a*^ All values reported in mg/L. MECs are presented for manogepix and MIC values for amphotericin B and the azoles.

### Other filamentous fungi

MEC/MIC parameters for manogepix, caspofungin, amphotericin B, posaconazole, and voriconazole against other filamentous fungi are shown in Table 4, and the MEC/MIC distributions are shown in Table 5. Similar to the results observed against *Aspergillus* and *Coccidioides* species, manogepix demonstrated potent *in vitro* activity against other filamentous fungi, including those with reduced *in vitro* susceptibility to other antifungals (e.g., *Scopulariopsis/Microascus* spp.) or are intrinsically resistant to certain azoles (*Rasamsonia argillacea* species complex and voriconazole, and *Purpureocillium lilacinum* and amphotericin B). Against these fungi, manogepix MECs ranged from ≤0.002 to 0.015 mg/L, with GM MEC values ranging from 0.003 to 0.005 mg/L and MEC_90_ values ranging from 0.008 to 0.015 mg/L. In contrast, the *in vitro* activity of caspofungin was reduced against *Scopulariopsis/Microascus* strains (MEC_90_ > 8 mg/L, GM MEC 0.454 mg/L; *P* < 0.0001 vs manogepix) and *P. lilacinum* (MEC_90_ 0.5 mg/L, GM MEC 0.176 mg/L; *P* < 0.0001 vs manogepix). As expected, amphotericin B, posaconazole, and voriconazole had limited activity against *Scopulariopsis/Microascus* strains, *Rasamsonia* strains were resistant to voriconazole (MIC range 4 to >16 mg/L), and *P. lilacinum* strains were resistant to amphotericin B (all MICs > 16 mg/L). Against the limited number of *Curvularia* spp. and *Exserohilum rostratum* strains tested, the *in vitro* activity of manogepix was reduced, with MECs ranging from 0.125 to 0.5 mg/L. Similar results were observed for caspofungin. Marked variability was observed for both manogepix and caspofungin against *Exophiala* spp. (manogepix MEC range ≤0.002 to >1 mg/L, caspofungin MEC range 0.25 to >8 mg/L).

**TABLE 4:** MEC/MIC parameters against other clinical strains of filamentous fungi (*n* = 132)^*a*^

Antifungal	Manogepix	Caspofungin	Amphotericin	Posaconazole	Voriconazole
*Scopulariopsis/Microascus* spp. (*n* = 50)					
Range	≤0.002–0.015	≤0.015 to >8	0.25 to >16	0.5 to >16	0.5 to >16
MEC/MIC50	≤0.002	0.25	4	4	16
MEC/MIC90	0.015	>8	16	>16	>16
GM MEC/MIC	0.003	0.454	3.03	5.74	9.58
Mode	≤0.002	>8	4	>16	16
*Rasamsonia argillacea* spp. complex (*n* = 51)					
Range	≤0.002–0.015	≤0.015 to >8	0.25–4	≤0.03–2	4 to > 16
MEC/MIC50	0.004	0.015	1	0.5	>16
MEC/MIC90	0.008	0.125	2	1	>16
GM MEC/MIC	0.005	0.028	0.95	0.51	>16
Mode	0.004	0.015	1	1	>16
*Purpureocillium lilacinum* (*n* = 12)					
Range	0.004–0.008	0.06–0.5	>16	0.25–0.5	0.25–1
MEC/MIC50	0.004	0.125	>16	0.25	0.25
MEC/MIC90	0.008	0.5	>16	0.5	1
GM MEC/MIC	0.004	0.176	>16	0.33	0.35
Mode	0.004	0.125	>16	0.25	0.25
*Curvularia* spp. (*n* = 8)					
Range	0.125	≤0.015–0.25	≤0.03–0.125	0.06–0.125	0.125 to >16
*Exophiala* spp. (*n* = 8)					
Range	≤0.002 to >1	0.25 to >8	0.03–1	≤0.03	0.06–1
*Exserohilum rostratum* (*n* = 3)					
Range	0.125–0.5	0.125–0.5	0.06–0.125	0.125–0.25	1–2

^*a*^ MECs reported for manogepix and caspofungin. MIC values reported for amphotericin B and azoles. All values reported in mg/L.

**TABLE 5:** MEC/MIC distributions against *Scopulariopsis/Microascus, Rasamsonia, Purpureocilliium, Curvularia, Exophiala,* and *Exserohilum rostratum* species^*a*^

MEC/MIC (mg/L)	≤0.002	0.004	0.008	0.015	0.03	0.06	0.125	0.25	0.5	1	2	4	8	16	>16
*Scopulariopsis/Microascus* spp. strains (*n* = 50)															
Manogepix	36	8	1	5											
Caspofungin				6	7	3	9	3	2		3	2	5	10	
Amphotericin								3	4	2	13	16	7	1	4
Posaconazole									4	4		14	6	3	19
Voriconazole									2	1	2	7	6	25	6
*Rasamsonia argillacea* species complex strains (*n* = 51)															
Manogepix	7	25	17	2											
Caspofungin				36	3	7	3							2	
Amphotericin								6	9	22	11	3			
Posaconazole					1	2	5	7	12	20	4				
Voriconazole												1	1	1	48
*Purpureocillium lilacinum* strains (*n* = 12)															
Manogepix		10	2												
Caspofungin						2	5	2	3						
Amphotericin															12
Posaconazole								7	5						
Voriconazole								7	4	1					
*Curvularia* spp. (*n* = 8)															
Manogepix							8								
Caspofungin				6			1	1							
Amphotericin					3	2	2	1							
Posaconazole						4	3		1						
Voriconazole							1		2	2	2				1
*Exophiala* spp. (*n* = 8)															
Manogepix	1		1	1		1					4				
Caspofungin								1						8	
Amphotericin					1		1	3		3					
Posaconazole					8										
Voriconazole						3	1	1	1	2					
*Exserohilum rostratum* (*n* = 3)															
Manogepix							1	1	1						
Caspofungin							1		2						
Amphotericin						2	1								
Posaconazole							1	2							
Voriconazole										2	1				

^*a*^ All values reported in mg/L. MECs are presented for manogepix and caspofungin, and MIC values are reported for amphotericin B and the azoles.

## DISCUSSION

Manogepix is the first-in-class member of a novel class of antifungal agents and targets the inositol acyltransferase protein Gwt1, which is a component of the GPI-anchored protein maturation pathway (20). The GPI pathway is important for trafficking and anchoring mannoproteins to the fungal cell membrane and wall (18, 19), as GPI-anchored mannoproteins serve as adhesions enabling fungi to adhere to cell surfaces within the host, allowing for colonization and infection (21). This agent is administered clinically to patients as the N-phosphonooxymethyl prodrug fosmanogepix (APX001). Following administration, this prodrug is rapidly converted to the active moiety by systemic phosphatases within the host (20, 37, 38).

Manogepix demonstrates potent and broad-spectrum antifungal activity. This includes activity against most *Candida* species (31, 37–40), including azole and echinocandin resistant strains, and the emerging pathogen *C. auris*, and *Cryptococcus neoformans* and *C. gattii* (29). *In vitro* activity has also been demonstrated against numerous pathogenic molds, such as *Aspergillus,* including azole-resistant strains*, Fusarium,* and *Scedosporium* species, and *Lomentospora prolificans* (18, 22, 23, 27, 41–43). Manogepix has also been shown to have activity against *Coccidioides* species, although the number of isolates tested against this dimorphic pathogen was small (30). The *in vitro* activity of manogepix has also translated into *in vivo* efficacy in several experimental models of invasive fungal infections, including those of candidiasis, cryptococcosis, aspergillosis, fusariosis, scedosporiosis, and coccidioidomycosis (22, 26, 27, 29–33). However, this agent has limited to no activity against *Candida krusei* (*Pichia kudriavsevii*) and similar species, such as *C. inconspicua* (*Pichia inconspicua*)*,* and *C. kefyr* (*Kluyveromyces marxianus*) (37). Mixed results have been reported against members of the order Mucorales. Some studies have reported no *in vitro* activity, while others have observed limited activity against *Rhizopus arrhizus* (18, 24, 40). However, improved *in vivo* activity has been observed when fosmanogepix was used in combination with liposomal amphotericin B compared to either agent alone in murine models of invasive aspergillosis, mucormycosis, and fusariosis (25).

The current study had several purposes. First, we confirmed the *in vitro* activity of manogepix against *A. fumigatus,* including azole-resistant strains harboring Cyp51 mutations, and also determined its potency against cryptic *Aspergillus* species. We also evaluated its activity against other filamentous fungi, including those that have reduced *in vitro* susceptibility to or are resistant to available antifungals. Finally, we determined the potency of manogepix against a larger number of *Coccidioides* isolates than what has previously been reported. Overall, manogepix demonstrated broad-spectrum and potent *in vitro* activity against most of the fungi used in this study. Against *Aspergillus* species, potent *in vitro* activity was observed against all species and strains tested, including azole-resistant strains, and cryptic species, such as *A. lentulus* and *A. calidoustus*, that have reduced susceptibility or frank *in vitro* resistance to extended-spectrum triazoles. The potency of manogepix was similar to that observed with caspofungin for most species, with the exception of *A. calidoustus,* against which the GM MEC for caspofungin was significantly higher. These results are similar to what others have reported for manogepix against *Aspergillus* species, including azole-resistant *A. fumigatus* and cryptic species, such as *A. lentulus, A. hortai, A. sydowii, A. tubingensis,* and members of *Aspergillus* section *Usti* (28, 44–46). Manogepix also demonstrated potent activity against *Coccidioides* species. Against this collection of 53 isolates, manogepix MEC values ranged from ≤0.002 to 0.03 mg/L, which is similar to what has previously been reported against a limited number of strains (MEC range 0.002 to 0.008 mg/L) (30). As noted previously, manogepix maintained activity against the single isolate that demonstrated high azole MICs.

Manogepix also demonstrated potent *in vitro* activity against other filamentous fungi that often demonstrate *in vitro* resistance or reduced susceptibility to clinically available agents. This included *Microascus/Scopulariopsis* species, which have reduced susceptibility or frank resistance to amphotericin B and the extended spectrum azoles (11, 47), members of the *Rasamsonia argillacea* species complex, which are intrinsically resistant to voriconazole and isavuconazole (13, 14), and *Purpureocillium lilacinum*, which is intrinsically resistant to amphotericin B (48). One study has also reported a low manogepix MEC value (0.008 mg/L) against a single *Microascus cirrosus* isolate, as well as low MECs against 12 isolates of the *R. argillaceae* species complex (range ≤0.008–0.016 mg/L) (38), which is consistent with the results of the current study. In contrast, higher manogepix MEC results were observed against a limited number of *Curvularia* and *Exserohilum rostratum* isolates, and marked variability was observed against *Exophiala* isolates in the current study. Others have reported consistently low manogepix MEC values against *Exophiala* isolates (range ≤0.008–0.016 mg/L) (38), although like in the current study, the number of strains included was small.

The results of this study are encouraging in that with a larger number of isolates confirm the findings of others that manogepix has potent *in vitro* activity against *Aspergillus* species, including azole-resistant strains, as well as *Coccidioides, Microascus,* and *Rasamsonia* species. Our results also add to the literature by demonstrating that manogepix also has potent activity against certain cryptic species of *Aspergillus* and *Purpureocillium lilacinum*, for which treatment options may be limited. Further studies are warranted to determine if the promising *in vitro* activity reported here translates into clinical efficacy in patients with these difficult-to-treat mycoses.

## MATERIALS AND METHODS

### Fungal isolates

Two hundred and fifty-seven clinical strains of different fungal species were used in this study. These included members of the genus *Aspergillus* (*n* = 72), *Coccidioides* spp. (*C. immitis* and *C. posadasii*; *n* = 53)*, Scopulariopsis/Microascuss* spp. (*n* = 50), *Rasamsonia argillacea* species complex (*n* = 51), *Purpureocillium lilacinum* (*n* = 12), *Curvularia* spp. (*n* = 8), *Exophiala* spp. (*n* = 8), and *Exserohilum rostratum* (*n* = 3). Species identification was performed by DNA sequence analysis of the partial β-tubulin, calmodulin, the translation elongation factor 1α genes, and/or the internal transcribed spacer region of rDNA (13, 49–54). Isolates were subcultured from frozen stocks onto potato flakes agar prior to *in vitro* testing.

### Antifungals

Manogepix was provided by Basilea Pharmaceutica (Basel, Switzerland), and caspofungin, amphotericin B, fluconazole, itraconazole, isavuconazole, voriconazole, and posaconazole were acquired from Sigma-Aldrich (St. Louis, MO, USA). Stock solutions of each antifungal were prepared in DMSO, with further dilutions prepared in RPMI with 0.165 M MOPS (buffered to pH 7.0, with phenol red, without sodium bicarbonate).

### *In vitro* susceptibility testing

Susceptibility testing was performed by broth dilution assay according to the methods published in the CLSI M38 standard (55). The endpoint used for manogepix (concentration range tested 0.002–4 mg/L) and caspofungin (0.002–8 mg/L) was the MEC, corresponding to the lowest concentration that resulted in morphologic changes (short, stubby, abnormally branched hyphae) after 24 to 48 h of incubation at 35°C. For amphotericin B, itraconazole, isavuconazole, posaconazole, and voriconazole (concentration range tested 0.03–16 mg/L for each), the MIC endpoint was the lowest concentration resulting in 100% inhibition of growth (visually clear well) after 48 h of incubation. For fluconazole, the MIC endpoint was the lowest concentration that resulted in a 50% reduction in growth compared to the growth control.

### Data analysis

Descriptive statistics, including MEC/MIC ranges, MEC/MIC values at which 50% and 90% of the isolates were inhibited (MEC/MIC_50_ and MEC/MIC_90_, respectively), geometric mean (GM) MEC/MIC, and modal MEC/MIC values were determined. The number of MEC/MIC values at each concentration level tested was also determined. Differences in GM MEC values between manogepix and caspofungin were assessed for significance by lognormal Welch’s *t*-test. A *P* value < 0.05 was considered significant.
